# The Cost-Effectiveness of Lifestyle Interventions for Preventing Diabetes in a Health Resource-Limited Setting

**DOI:** 10.1155/2020/7410797

**Published:** 2020-04-13

**Authors:** Jing Ma, Xu Wan, Bin Wu

**Affiliations:** ^1^Department of Endocrinology, Ren Ji Hospital, School of Medicine, Shanghai Jiaotong University, China; ^2^Medical Decision and Economic Group, Department of Pharmacy, Ren Ji Hospital, School of Medicine, Shanghai Jiaotong University, China

## Abstract

**Aims:**

Type 2 diabetes mellitus (T2DM) is a health challenge in China, and the economic outcomes of lifestyle intervention are critically important for policymakers. This study estimates the lifetime economic outcomes of lifestyle intervention among the prediabetic population in the Chinese context.

**Methods:**

We developed a mathematical model to compare the cost-effectiveness of lifestyle intervention and no prevention in the prediabetic population. Efficacy and safety, medical expenditure, and utility data were derived from the literature, which was assigned to model variables for estimating the quality-adjusted life-years (QALYs) and costs as well as incremental cost-effectiveness ratios (ICERs). The analysis was conducted from the perspective of Chinese healthcare service providers. One-way and probabilistic sensitivity analyses were performed.

**Results:**

Compared with no prevention, lifestyle intervention averted 9.53% of T2DM, which translated into an additional 0.52 QALYs at a saved cost of $700 by substantially reducing the probabilities of macro- and microvascular diseases. This finding indicated that lifestyle intervention was a dominant strategy. The sensitivity analyses showed the model outputs were robust.

**Conclusions:**

Lifestyle intervention is a very cost-effective alternative for prediabetic subjects and worth implementing in the Chinese healthcare system to reduce the disease burden related to T2DM.

## 1. Introduction

With the largest population, the prevalence of diabetes in China is 11.6% of adults [1, 2]. It has been shown that all-age disability-adjusted life-years (DALYs) of diabetes were increased by 24.4% (95% CI: 22.7–26.2) from 1990 to 2016 according to the Global Burden of Disease Study [3]. The cost burden of diabetes increased about 100 times in 14 years [4, 5]. Type 2 diabetes mellitus (T2DM) occupied around 90%-95% of the disease.

Lifestyle intervention has been advocated to decrease the risk of T2DM in prediabetic subjects, which could reduce 3% absolute risk compared with no prevention [6–8]. The Da Qing Diabetes Prevention Study found that a 6-year lifestyle intervention program for those with impaired glucose tolerance (IGT) reduced the 17.3% cumulative incidence of diabetes (hazard ratio (HR): 0.55, 95% CI: 0.40-0.76) during the 23-year follow-up period [9]. Chinese diabetes society encouraged lifestyle intervention to decrease the risk of diabetes in general and high-risk population [10]. T2DM has become a leading health challenge in China. The cost-effectiveness of prevention strategies is emergent for informing health policy decision makers. Economic analysis in North America and Europe shows that lifestyle intervention is cost-effective relative to no prevention [11]. However, few evidences are currently available for Asian countries. Because the economic results from other countries may not be generalizable to the Chinese context, we sought to determine the anticipated health economic outcomes for lifestyle intervention in high-risk population and compare it to those with no prevention in the Chinese healthcare setting.

## 2. Methods

### 2.1. Model Overview

The economic analysis of primary prevention included the high-risk population of T2DM, who were initially assigned to lifestyle intervention or no prevention. The lifestyle intervention included the diet and exercise interventions. Based on the Da Qing Diabetes Prevention Study, the diet intervention was designed to produce weight loss in those who were overweight or obese and to reduce simple carbohydrate and alcohol intake in people of normal weight. The exercise intervention was designed to increase the leisure time spent doing physical activity [9].

**Figure 1:**
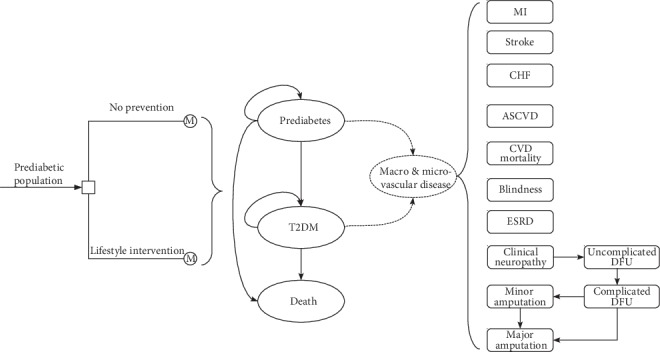
Schematic of type 2 diabetes mellitus prevention model.

The analysis was carried out by using a mathematical model, which could annually track the T2DM progression from prediabetes (Figure 1). Once the patients developed T2DM, the disease course would be projected by using the Chinese Outcomes Model for T2DM (COMT) [8, 12, 13]. This validated diabetes policy analysis model would track simultaneously several critical diabetic macro- and microvascular complications for one hypothetical T2DM patient, including myocardial infarction (MI), congestive heart failure (CHF), cardiovascular disease (CVD), stroke, blindness, end-stage renal disease (ESRD), clinical neuropathy, foot ulcer, and minor and major amputation. One patient could incur different complications at the same time. The all-cause mortality would be adjusted based on the treatment effect and disease status. Each diabetic complication is an independent submodel that was integrated with the COMT model. The transition probabilities of the model were estimated according to the latest Risk Equations for Complications of Type 2 Diabetes (RECODe) [14], which is adjusted validated based on the Chinese patient characteristics of T2DM. The details about the model development and validation could be found in our previous report [12]. During the model simulation, interconnectivity and interaction among submodels of individual complication were permitted to allow the complication risks to be updated by using tracker parameters. The clinical and demographic characteristics of the hypothetical cohorts with T2DM were used for determining the annual disease progression: sex, age, smoking status, systolic blood pressure (SBP), glycated haemoglobin (HbA1c), total and high-density lipoprotein (HDL) cholesterol levels, serum creatinine, urine albumin : creatinine ratio, history of cardiovascular disease, and use of antihypertensive, anticoagulant medications, statin, and oral diabetes medication. During the simulation, risk parameters might be updated based on the treatment transition, thereby resulting in the likelihood of complication incidence. HbA1c, SBP, and cholesterol would worsen over time. More details about the model process could be found in our previous work [12]. The design of the model was the same for lifestyle intervention and no prevention strategies, with only risks of developing complications adjusted by the use of the different intervention.

Lifetime health such as complication probabilities life-year (LY) and quality-adjusted life-year (QALY) and costs would be processed in the current analysis. Costs and QALY were annually discounted at 5%, according to data released by the Chinese health economic recommendation [15]. Cost-effectiveness was defined if the incremental cost-effectiveness ratios (ICERs) were less than the per capita gross domestic product (GDP) of China in 2019 ($10,276) [15].

### 2.2. Patient Profile and Disease Progression

Based on the Chinese practice, individuals with high risk for diabetes were screened to confirm the glycemic state by oral glucose tolerance test [16]. The population characteristic profile was assumed to be similar to the Da Qing Diabetes Prevention Study, which enrolled 577 Chinese adults with confirmed IGT whose baseline characteristics were shown as follows: 45 years old, 54% of male, BMI 25.8 kg/m^2^, SBP 132.9 mmHg, DBP 87.6 mmHg, FPG 5.57 mmol/L, 2hPG 9.0 mmol/L, and current smoker 41.2% [9]. This trial showed that the incidence of diabetes was 72.6% (68.4-76.8) in the intervention group versus 89.9% in the control group (84.9-94.9; HR 0.55, 95% CI 0.40-0.76; *p* = 0.001) [9]. The Kaplan-Meier curves of developing diabetes in a 23-year follow-up were fitted by using the Weibull survival function *S* (*t*) = exp(–*αt*^*β*^), whose parameters are shown in Table 1. Patients with T2DM would incur the macro- and microvascular disease, whose risks in COMT model were estimated by using the adjusted Risk Equations for Complications of Type 2 Diabetes (RECODe) [14]. For those without diabetes, the risks of the macro- and microvascular disease were estimated by multiplying the absolute risks in diabetic patients and the relative risks of subjects without diabetes versus diabetic patients, which were derived from the published literature [17–24]. The natural mortality rates were based on the 2009 Chinese life tables reported by the World Health Organization (WHO) [25].

### 2.3. Costs and Utility

The current study was presented from the point of the Chinese healthcare provider. Therefore, medical costs were the focus in the model (Table 1). All data were shown in the 2019 US dollar (1 US dollar = CNY ¥ 6.9). Because the lifestyle intervention for high-risk population and diabetic population was comparable [9, 16], we assumed that the cost related to lifestyle intervention was similar with an economic study in China [26], which reported that the annual cost of diabetic education was $134.0 per patient. This fee would be used for paying the works of practice nurses, who would educate and prescribe a lifestyle prescription for patient. The annual costs of medicine and glucose testing strips were originated from a large scale population-based study [27]. The costs of inpatient and outpatient visits due to vascular complications were extracted directly from publications [4, 28–32]. The expense of severe hypoglycemic events was derived from a Chinese costing study, which included 275 patients who incurred hypoglycemic episode [33]. The target population has been confirmed with prediabetes, so the cost related to an oral glucose tolerance test was not included in this analysis.

Utility scores were collected from a recent study. It involved 289 T2DM patients in China. The utility scores of neuropathy, heart disease, and cerebrovascular disease were analyzed by using EQ-5D-5L [34].

### 2.4. Sensitivity Analysis

One-way and probabilistic sensitivity analyses (PSA) were used to analyze the potential drivers of economic outcomes. The range of the ICERs between low and high values was shown in Table 1. Ranges from 75% to 125% of base case values were assumed when there was no available data. In PSA with the second-order Monte-Carlo simulations (1000 iterations), all model variables were attached by probability distributions. The probability, proportions, and utility scores followed beta distribution, a triangle distribution was modeling for cost, and normal distribution was for hazard ratio and patient characteristics. If there was no reported data, we assumed 25% of the base case value to be the standard error. Cost-effectiveness acceptability curve (CEAC) was generated based on the results of PSA.

## 3. Results

### 3.1. Base Case Analysis

In comparison with no prevention, the lifestyle intervention reduced the 9.53% cumulative probabilities of diabetes, which resulted in mean additional benefits in life expectancy and QALY of 0.82 years and 0.52 QALYs, respectively, at saved total mean costs of $700 over a lifetime period (Table 2). The ICER between lifestyle intervention and no intervention was $ -1339 per QALY gained (dominant strategy).

### 3.2. Sensitivity Analysis

**Figure 2:**
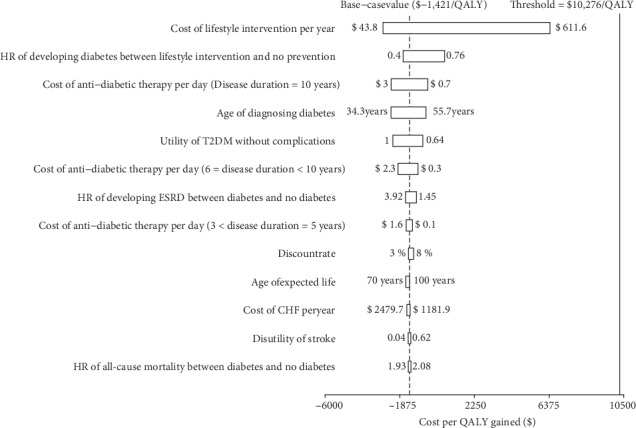
Tornado diagram representing the cost-effectiveness in one-way sensitivity analysis for lifestyle interventions versus no prevention. The width of the bars represents the range of the results when the variables were changed.

The model was more sensitive to the cost of the lifestyle intervention and the hazard ratio of developing diabetes between lifestyle intervention and no intervention because they were found to have a substantial impact on ICER. The remainder sensitive variables, such as the cost and utility of managing diabetes and its complications, had a medium or small impact (Figure 2). However, none of the adjustments of parameters could lead the ICERs to be higher than $10,276/QALY.

**Figure 3:**
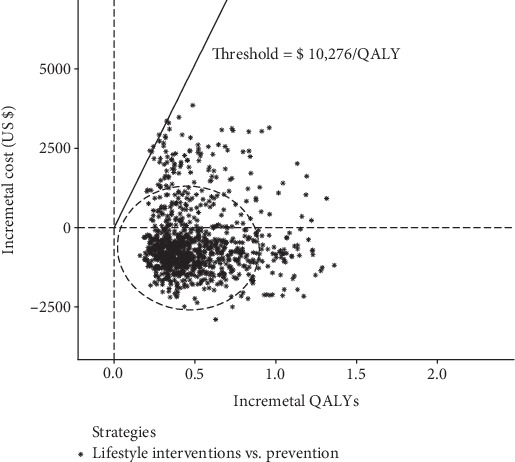
The probabilistic results of the incremental cost-effectiveness difference of lifestyle interventions versus no prevention. The dashed ellipses surround 95% of the estimates.

**Figure 4:**
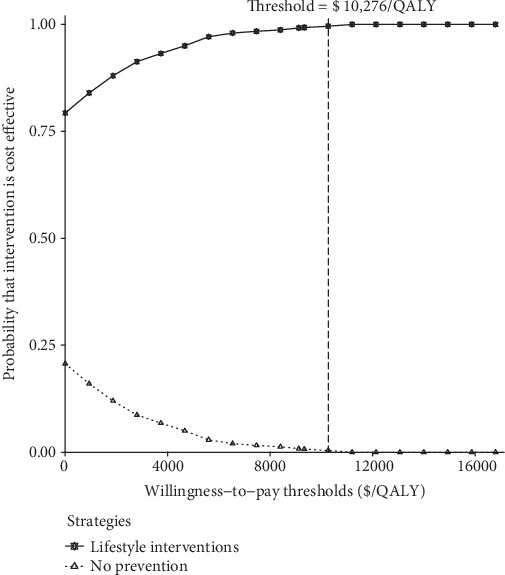
Cost-effectiveness acceptability curve for lifestyle interventions versus no prevention.

In the probabilistic sensitivity analyses (Figure 3), the cost per additional QALY gained for lifestyle intervention over no prevention was $ -1578 (95% CI: $ -2858 to $ -821). At an acceptable ICER of $10,276 (GDP per capita of China in 2019), over 95% probabilities of cost-effectiveness were produced by lifestyle interventions according to the acceptability curve (Figure 4).

## 4. Discussion

Reports of the health benefits of lifestyle intervention in clinical trials excite both endocrinologists and prediabetic subjects. However, lifestyle intervention accompanies with a considerable increase in healthcare costs compared with no prevention, which is of concern to payers. The need for the precise economic evaluation of implementing lifestyle intervention in the Chinese context is becoming urgent. It is the first economic evaluation that estimated, in Chinese subjects with prediabetes, the initiation of lifestyle intervention was associated with improvements in length and quality of life and saving money by preventing or delaying T2DM. Sensitivity analysis showed that economic outcomes were not sensitive to the adjustments of the model inputs and assumptions and kept the life intervention strategy cost-effective. This finding is emphasized by the involvement of the COMT model, which is a well-established model for surrogate endpoints such as blood glucose, blood pressure, and lipid profiles in the Chinese population. Our analysis indicated that lifestyle intervention is worth implementing in the Chinese healthcare system as it is considered to be very cost-saving. This study presents a key step in the Chinese diabetes prevention policy: whether to aim interventions at those with the most attractive ICERs (<per capita GDP).

Our findings are comparable with those previously published economic evaluations. One systematic review summarized that those cost evaluations of lifestyle interventions in high-risk diabetic subjects are cost-effective [35]. The ICERs of lifestyle intervention programs against no prevention varied from cost-saving to $175,754/QALY with a median value of £9,793/QALY. However, there are few studies in low-income and middle-income regions. Literature reveals that lifestyle intervention for diabetes was cost-effective among high-risk Indian [36]. One Chinese study also demonstrated that appropriate lifestyle interventions for those with IGT are cost-saving in China [37]. However, this study reported by Liu and colleagues did not separately report the economic outcome of lifestyle intervention in high-risk population due to their study goal. Differences in study design, model structure, assumptions, and inputs contribute to the variation of ICERs in these economic reports.

This study has some limitations. Firstly, lifestyle interventions led to notable improvements in the risk profiles of cardiovascular disease in normal adults, which might further decrease CVD risk [38]. By using a conservative approach to measure the health outcomes of lifestyle modification, we did not consider the health benefits in the population who did not develop diabetes and other types of disease (e.g., obesity-related cancers or dementia), which might underestimate the economic outcomes of lifestyle intervention. Secondly, due to the absence of solid evidence in the Eastern population, the current analysis did not include metformin as a competing alternative for preventing diabetes, which also showed cost-effectiveness in other regions [35]. Thirdly, some of the clinical data obtained from literature might not be related to Chinese data. However, since the sensitivity analysis is robust, these may be taken into account. Lastly, since the analysis is based on the Da Qing Diabetes Prevention Study that only enrolled subjects with IGT, the economic outcome may be affected if prediabetic subjects differ significantly from it, such as the subjects with impaired fasting glucose, whose aetiologies seem to differ from IGT [39]. The sensitivity analyses showed that ICER is comparatively sensitive to the effectiveness of lifestyle interventions, although cost-effective might be plausible for Chinese given our reading of the current study. Future research should focus on establishing individualized preventive strategies based on the differently prediabetic states [40]. Nevertheless, as the results of this analysis address the common health issues of diabetes in China, we believe these results can be an important reference point for Chinese policymakers.

In summary, according to the analysis in our study, lifestyle intervention is a cost-effective alternative for prediabetic subjects and worth implementing in the Chinese healthcare system. The preventive program needs to be tailored in order to further optimize the allocation of health resources in future research.

## Figures and Tables

**Table 1:** Key model inputs.

Parameters	Expected values	Ranges	Source
Clinical data			
Weibull function parameters			
No prevention	*α* = 0.177, *β* = 0.8551	NA	[9]
Life interventions	*α* = 0.0749, *β* = 0.96	NA	[9]
RR of diabetes of life interventions vs. no prevention	0.55	0.4-0.76	[9]
RR of MI of T2DM vs. no diabetes	2	1.83-2.19	[17]
RR of stroke of T2DM vs. no diabetes	2.27	1.95-2.65	[17]
RR of CVD death of T2DM vs. no diabetes	2.38	1.45-3.92	[17]
RR of CHF of T2DM vs. no diabetes	2.5	2.3-2.7	[18, 19]
RR of blindness of T2DM vs. no diabetes	1.43	1.04-2.32	[21, 22]
RR of clinical neuropathy of T2DM vs. no diabetes	4.60	3.18-6.02	[23, 24]
RR of chronic kidney disease of T2DM vs. no diabetes	2.38	1.45-3.92	[20]
Costs (US $)^#^			
Life interventions	134.0	43.8-611.6	[26]
Antidiabetic therapy per day (disease duration ≤ 2 years)	0.5	0.1-1.2	[27]
Antidiabetic therapy per day (3 < disease duration ≤ 5 years)	0.8	0.1-1.6	[27]
Antidiabetic therapy per day (6 ≤ disease duration < 10 years)	1.2	0.3-2.3	[27]
Antidiabetic therapy per day (disease duration≥10 years)	1.8	0.7-3	[27]
MI hospitalization per event	6955.0	6128-7782	[4, 28–30]
Care after MI per year	429.0	271.9-586.1	[4, 28–30]
Stroke hospitalization per event	2708.6	2058-4463.6	[4, 28–30]
Care after stroke per year	477.5	420.1-780	[4, 28–30]
CHF per year	1420.3	1181.9-2479.7	[4, 28–30]
ESRD per year	13,003.0	12,391.3-13,724.6	[31]
Blindness per year	1546.8	1347.5-1746	[4, 28–30]
Clinical neuropathy per month	57.4	24.7-95.5	[32]
Uncomplicated DFU per event	71.7	0-213	[32]
Complicated DFU per event	2160.3	1157.2-2713.8	[32]
Minor amputation per event	3124.6	2039.7-4746.8	[32]
Major amputation per event	4728.3	2808.3-7289.6	[32]
Care after major amputation per month	318.5	0-565.9	[32]
Severe hypoglycemia per event	534.4	400.8-667.9	[33]
Utility values			
T2DM without complications	0.876	0.736-1	[34]
Utility decrements			
MI hospitalization for one month	1.000	0.236-1	[34]
MI after discharge	0.236	0.026-0.446	[34]
Stroke hospitalization for one month	1.000	0.326-1	[34]
Stroke after discharge	0.326	0.036-0.616	[34]
CHF	0.236	0.026-0.446	[34]
ESRD	0.400	0.19-0.61	[4, 28–32]
Blindness	0.157	0.007-0.307	[4, 28–32]
Clinical neuropathy	0.185	0.015-0.355	[34]
Uncomplicated DFU	0.250	0.213-0.287	[4, 28–32]
Complicated DFU	0.300	0.165-0.435	[4, 28–32]
Minor amputation	0.320	0.204-0.436	[4, 28–32]
Major amputation	0.380	0.264-0.496	[4, 28–32]

Abbreviations: NA: not applicable; RR: relative risk; T2DM: type 2 diabetes mellitus; DFU: diabetic foot ulcers; MI: myocardial infarction; CHF: congestive heart failure; CVD: cardiovascular disease; ESRD: end-stage renal disease. ^#^All cost data were shown in the 2019 US dollar (1 US dollar = CNY ¥ 6.9).

**Table 2:** Base case results for lifestyle interventions compared to no prevention.

Outcomes	No prevention	Lifestyle interventions	Difference^∗^
Cumulative probabilities of diabetes	93.57%	84.04%	-9.53%
Cumulative probabilities of complications			
MI	10.56%	9.93%	-0.63%
Stroke	24.17%	22.62%	-1.55%
CHF	14.89%	13.71%	-1.18%
ESRD	4.61%	4.22%	-0.39%
Blindness	5.79%	5.66%	-0.13%
Clinical neuropathy	21.28%	20.25%	-1.03%
Minor amputation	12.21%	10.55%	-1.66%
Major amputation	9.22%	7.94%	-1.28%
Total QALY	13.03	13.55	0.52
Total LY	27.87	28.68	0.82
Total cost (US $)^#^	11,390	10,689	-700
ICER (US $/QALY)	NA	Dominant	

^∗^Compared with the control strategy (no prevention). ^#^All cost data were shown in the 2019 US dollar (1 US dollar = CNY ¥ 6.9).

## Data Availability

No additional data are available.

## Additional Points

*Provenance and Peer Review*. Not commissioned; externally peer reviewed.

## References

[1] Xu Y., Wang L., He J. (2013). Prevalence and control of diabetes in Chinese adults. *JAMA*.

[2] Wang L., Gao P., Zhang M. (2017). Prevalence and ethnic pattern of diabetes and prediabetes in China in 2013. *Journal of the American Medical Association*.

[3] GBD 2016 DALYs and HALE Collaborators (2017). Global, regional, and national disability-adjusted life-years (DALYs) for 333 diseases and injuries and healthy life expectancy (HALE) for 195 countries and territories, 1990-2016: a systematic analysis for the Global Burden of Disease Study 2016. *The Lancet*.

[4] Wang H., Lin X., Zhang Z. (2015). The economic burden of inpatients with type 2 diabetes: a case study in a Chinese hospital. *Asia-Pacific Journal of Public Health*.

[5] Chan J. C. N., Zhang Y., Ning G. (2014). Diabetes in China: a societal solution for a personal challenge. *The Lancet Diabetes and Endocrinology*.

[6] Haw J. S., Galaviz K. I., Straus A. N. (2017). Long-term sustainability of diabetes prevention approaches: a systematic review and meta-analysis of randomized clinical trials. *JAMA Internal Medicine*.

[7] Galaviz K. I., Weber M. B., Straus A., Haw J. S., Narayan K. M. V., Ali M. K. (2018). Global diabetes prevention interventions: a systematic review and network meta-analysis of the real-world impact on incidence, weight, and glucose. *Diabetes Care*.

[8] Nian H., Wan X., Ma J., Jie F., Wu B. (2020). Economic evaluation of dapagliflozin versus metformin in Chinese patients whose diabetes is inadequately controlled with diet and exercise. *Cost Effectiveness and Resource Allocation*.

[9] Li G., Zhang P., Wang J. (2014). Cardiovascular mortality, all-cause mortality, and diabetes incidence after lifestyle intervention for people with impaired glucose tolerance in the Da Qing Diabetes Prevention Study: a 23-year follow-up study. *The Lancet Diabetes and Endocrinology*.

[10] Chinese Diabetes Society (2014). 2013 China guideline for prevention and treatment of type 2 diabetes. *Chinese Journal of Endocrinology and Metabolism*.

[11] Alouki K., Delisle H., Bermúdez-Tamayo C., Johri M. (2016). Lifestyle interventions to prevent type 2 diabetes: a systematic review of economic evaluation studies. *Journal of Diabetes Research*.

[12] Wu B., Ma J., Zhang S., Zhou L., Wu H. (2018). Development and validation of a health policy model of type 2 diabetes in Chinese setting. *Journal of Comparative Effectiveness Research*.

[13] Li T., Wan X., Ma J., Wu B. (2018). Cost-effectiveness of primary prevention with statin treatment for Chinese patients with type 2 diabetes. *Advances in Therapy*.

[14] Basu S., Sussman J. B., Berkowitz S. A., Hayward R. A., Yudkin J. S. (2017). Development and validation of Risk Equations for Complications of type 2 Diabetes (RECODe) using individual participant data from randomised trials. *The Lancet Diabetes and Endocrinology*.

[15] Wang X., Wang Z.-F., Xie Y.-M. (2015). Guideline for postmarketing Chinese medicine pharmacoeconomic evaluation. *Chinese Journal of Integrative Medicine*.

[16] Luo Y., Paul S. K., Zhou X. (2017). Rationale, design, and baseline characteristics of Beijing prediabetes reversion program: a randomized controlled clinical trial to evaluate the efficacy of lifestyle intervention and/or pioglitazone in reversion to normal glucose tolerance in prediabetes. *Journal of Diabetes Research*.

[17] The Emerging Risk Factors Collaboration (2010). Diabetes mellitus, fasting blood glucose concentration, and risk of vascular disease: a collaborative meta-analysis of 102 prospective studies. *The Lancet*.

[18] Nichols G. A., Gullion C. M., Koro C. E., Ephross S. A., Brown J. B. (2004). The incidence of congestive heart failure in type 2 diabetes: an update. *Diabetes Care*.

[19] Lehrke M., Marx N. (2017). Diabetes mellitus and heart failure. *The American Journal of Cardiology*.

[20] Fox C. S., Larson M. G., Leip E. P., Culleton B., Wilson P. W., Levy D. (2004). Predictors of new-onset kidney disease in a community-based population. *JAMA*.

[21] Chong E. W., Lamoureux E. L., Jenkins M. A., Aung T., Saw S.-M., Wong T. Y. (2009). Sociodemographic, lifestyle, and medical risk factors for visual impairment in an urban Asian population: the Singapore Malay eye study. *Archives of Ophthalmology*.

[22] Cui Y., Zhang L., Zhang M. (2017). Prevalence and causes of low vision and blindness in a Chinese population with type 2 diabetes: the Dongguan Eye Study. *Scientific Reports*.

[23] Lu B., Hu J., Wen J. (2013). Determination of peripheral neuropathy prevalence and associated factors in Chinese subjects with diabetes and pre-diabetes - ShangHai Diabetic neuRopathy Epidemiology and Molecular Genetics Study (SH-DREAMS). *PloS One*.

[24] Tapp R. J., Shaw J. E., de Courten M. P., Dunstan D. W., Welborn T. A., Zimmet P. Z. (2003). Foot complications in type 2 diabetes: an Australian population-based study. *Diabetic Medicine*.

[25] WHO, Life expectancy. https://www.who.int/gho/mortality_burden_disease/life_tables/en/.

[26] Guo X., Pavika J., YU C. (2012). Patient education for type 2 diabetics is cost-effective in China. *Chinese Journal of Dlabetes Mellitus*.

[27] Yang W., Zhao W., Xiao J. (2012). Medical care and payment for diabetes in China: enormous threat and great opportunity. *PLoS One*.

[28] Li T., Liu M., Ben H., Xu Z., Zhong H., Wu B. (2015). Clopidogrel versus aspirin in patients with recent ischemic stroke and established peripheral artery disease: an economic evaluation in a Chinese setting. *Clinical Drug Investigation*.

[29] Wu B., Wan X., Ma J. (2018). Cost-effectiveness of prevention and management of diabetic foot ulcer and amputation in a health resource-limited setting. *Journal of Diabetes*.

[30] Shao H., Zhai S., Zou D. (2017). Cost-effectiveness analysis of dapagliflozin versus glimepiride as monotherapy in a Chinese population with type 2 diabetes mellitus. *Current Medical Research and Opinion*.

[31] Gu S., Mu Y., Zhai S., Zeng Y., Zhen X., Dong H. (2016). Cost-effectiveness of dapagliflozin versus acarbose as a monotherapy in type 2 diabetes in China. *PLoS One*.

[32] Wu B., Zhang S., Lin H., Mou S. (2018). Prevention of renal failure in Chinese patients with newly diagnosed type 2 diabetes: a cost-effectiveness analysis. *Journal of Diabetes Investigation*.

[33] Ya-Ming Z., Wu J., Xie K. (2012). Incidence and cost of hypoglycemia episode in patients with type 2 diabetes mellitus (T2DM). *Chinese Rural Health Service Administration*.

[34] Pan C.-W., Sun H.-P., Zhou H.-J. (2016). Valuing health-related quality of life in type 2 diabetes patients in China. *Medical Decision Making*.

[35] Roberts S., Barry E., Craig D., Airoldi M., Bevan G., Greenhalgh T. (2017). Preventing type 2 diabetes: systematic review of studies of cost-effectiveness of lifestyle programmes and metformin, with and without screening, for pre-diabetes. *BMJ Open*.

[36] Ramachandran A., Snehalatha C., Yamuna A., Mary S., Ping Z. (2007). Cost-effectiveness of the interventions in the primary prevention of diabetes among Asian Indians: within-trial results of the Indian Diabetes Prevention Programme (IDPP). *Diabetes Care*.

[37] Liu X., Li C., Gong H. (2013). An economic evaluation for prevention of diabetes mellitus in a developing country: a modelling study. *BMC Public Health*.

[38] Zhang X., Devlin H. M., Smith B. (2017). Effect of lifestyle interventions on cardiovascular risk factors among adults without impaired glucose tolerance or diabetes: a systematic review and meta-analysis. *PLoS One*.

[39] Faerch K., Borch-Johnsen K., Holst J. J., Vaag A. (2009). Pathophysiology and aetiology of impaired fasting glycaemia and impaired glucose tolerance: does it matter for prevention and treatment of type 2 diabetes?. *Diabetologia*.

[40] Roberts S., Craig D., Adler A., McPherson K., Greenhalgh T. (2018). Economic evaluation of type 2 diabetes prevention programmes: Markov model of low- and high-intensity lifestyle programmes and metformin in participants with different categories of intermediate hyperglycaemia. *BMC Medicine*.

